# Prenatal alcohol exposure alters mRNA expression for stress peptides, glucocorticoid receptor function and immune factors in acutely stressed neonatal brain

**DOI:** 10.3389/fnins.2023.1203557

**Published:** 2023-06-23

**Authors:** Chaselyn D. Ruffaner-Hanson, Annette K. Fernandez-Oropeza, Melody S. Sun, Kevin K. Caldwell, Andrea M. Allan, Daniel D. Savage, C. Fernando Valenzuela, Shahani Noor, Erin D. Milligan

**Affiliations:** Department of Neurosciences, University of New Mexico School of Medicine, Albuquerque, New Mexico

**Keywords:** prenatal alcohol exposure, TLR4, amygdala, maternal separation, stress, FKBP5, Gas5, glucocorticoid receptor

## Abstract

**Background:**

The amygdala, hippocampus and hypothalamus are critical stress regulatory areas that undergo functional maturation for stress responding initially established during gestational and early postnatal brain development. Fetal alcohol spectrum disorder (FASD), a consequence of prenatal alcohol exposure (PAE), results in cognitive, mood and behavioral disorders. Prenatal alcohol exposure negatively impacts components of the brain stress response system, including stress-associated brain neuropeptides and glucocorticoid receptors in the amygdala, hippocampus and hypothalamus. While PAE generates a unique brain cytokine expression pattern, little is known about the role of Toll-like receptor 4 (TLR4) and related proinflammatory signaling factors, as well as anti-inflammatory cytokines in PAE brain stress-responsive regions. We hypothesized that PAE sensitizes the early brain stress response system resulting in dysregulated neuroendocrine and neuroimmune activation.

**Methods:**

A single, 4-h exposure of maternal separation stress in male and female postnatal day 10 (PND10) C57Bl/6 offspring was utilized. Offspring were from either prenatal control exposure (saccharin) or a limited access (4 h) drinking-in-the-dark model of PAE. Immediately after stress on PND10, the hippocampus, amygdala and hypothalamus were collected, and mRNA expression was analyzed for stress-associated factors (CRH and AVP), glucocorticoid receptor signaling regulators (GAS5, FKBP51 and FKBP52), astrocyte and microglial activation, and factors associated with TLR4 activation including proinflammatory interleukin-1β (IL-1β), along with additional pro- and anti-inflammatory cytokines. Select protein expression analysis of CRH, FKBP and factors associated with the TLR4 signaling cascade from male and female amygdala was conducted.

**Results:**

The female amygdala revealed increased mRNA expression in stress-associated factors, glucocorticoid receptor signaling regulators and all of the factors critical in the TLR4 activation cascade, while the hypothalamus revealed blunted mRNA expression of all of these factors in PAE following stress. Conversely, far fewer mRNA changes were observed in males, notably in the hippocampus and hypothalamus, but not the amygdala. Statistically significant increases in CRH protein, and a strong trend in increased IL-1β were observed in male offspring with PAE independent of stressor exposure.

**Conclusion:**

Prenatal alcohol exposure creates stress-related factors and TLR-4 neuroimmune pathway sensitization observed predominantly in females, that is unmasked in early postnatal life by a stress challenge.

## Introduction

Maternal consumption of alcohol during pregnancy results in a broad constellation of outcomes known as fetal alcohol spectrum disorder (FASD) and includes central nervous system (CNS) deficits manifesting as cognitive, behavioral and emotional dysregulation (Moore and Xia, 2021). Alcohol can interact with CNS systems such as the hypothalamic–pituitary–adrenal (HPA) axis that regulate stress responses. Published data show that, as a consequence of alcohol consumption during pregnancy, increased maternal glucocorticoids lead to detrimental exposure levels to the fetus (O'Connor et al., 1998; Przybycien-Szymanska et al., 2011). Further, alcohol can alter the expression of corticotropin-releasing hormone (CRH), a factor that initiates HPA activation in response to alcohol or stress, and is released within several key brain regions involved in stress regulation such as the periventricular nucleus within the hypothalamus (O'Connor et al., 1998), the amygdala, and the hippocampus (Tsigos et al., 2000; Charney, 2004; Zhou and Fang, 2018). In addition to fetal exposure to deleterious levels of maternal stress hormones, maternal consumption of alcohol poses direct developmental risks to the fetus, as alcohol is capable of crossing the placenta to interact directly with ongoing fetal CNS maturation including HPA axis regulation (Gabriel et al., 1998). That is, prenatal alcohol exposure (PAE) results in elevated glucocorticoids in fetal blood and acts in brain, altering glucocorticoid receptor expression and signaling in the hypothalamus, amygdala and hippocampus (Caldwell et al., 2014; Lam et al., 2018; Lu et al., 2018; Raineki et al., 2019). Importantly, the consequence of elevated glucocorticoid levels during CNS maturation results in reduced CNS neural development and myelination (De Kloet et al., 1988). While the changes and the impact of initial biochemical factors/receptors resulting from direct alcohol exposure from maternal alcohol consumption differ from maternal stress, the downstream consequences of fetal exposure to elevated glucocorticoids (Gabriel et al., 1998; Lan et al., 2017) converge resulting in an enduring dysregulation of the brain stress-response and HPA axis in offspring (Rash et al., 2016; Gartstein and Skinner, 2018; McGowan and Matthews, 2018).

A large body of evidence demonstrates an extensive interface of the neuro-immune-endocrine systems, with bi-directional communication and regulation (Elenkov et al., 2000; Haddad et al., 2002) through the actions of common ligands including cytokines/chemokines and their receptors in brain regions such as, but not limited to, the hypothalamus, amygdala and hippocampus. While glucocorticoids inhibit the production of several potent proinflammatory cytokines including interleukin (IL)-1β (Páez Pereda et al., 1996; Goujon et al., 1997; Plagemann et al., 1998), conversely, brain proinflammatory cytokines induce HPA activation through multiple mechanisms including reducing glucocorticoid receptor sensitivity (Pariante et al., 1999; Daun et al., 2000). More recent studies have extended the role of the neuro-immune-endocrine interface in underlying CNS dysregulation as a consequence of PAE (Bodnar et al., 2016), emphasizing the critical influence that a variety of cytokines exert in the developing CNS, particularly in offspring following PAE.

Prenatal alcohol exposure negatively impacts the delicate balance of neuroimmune factors. Increases in glial (microglia and astrocytes) activation, proinflammatory cytokines IL-1β and tumor necrosis factor-a (TNF-α), and the chemokine known as C–C motif chemokine ligand-2 (CCL2) are observed in the brain from offspring with PAE, along with decreases in the anti-inflammatory cytokine, interleukin-10 (IL-10; Drew et al., 2015; Topper et al., 2015) and in the spinal cord following peripheral nerve damage (Noor et al., 2017, 2020; Sanchez et al., 2017, 2019). Additionally, PAE female rat pups at postnatal day (PND) 8 reveal IL-1β and TNF-α protein are decreased in the hypothalamus and increased in the hippocampus (Bodnar et al., 2016). However, it remains poorly understood how altered early-life neuroendocrine-immune function may impact factors that initiate the brain stress response system (i.e., CRH) in neonatal brain regions where glucocorticoid receptors are expressed.

Given PAE exerts strong influences on the brain neuroimmune-neuroendocrine interface, the goal of the current study was to examine whether PAE alters early-life transcriptional (mRNA) expression of CRH and arginine vasopressin (AVP), which play critical roles in initiating the HPA stress responses, in addition to basal and stress-induced neuroimmune factors generated by stimulation of the Toll-like Receptor 4 (TLR4). Activated TLR4 results in a proinflammatory cellular signaling cascade of cytokines, chemokines, and transcription factors (Chen and Jiang, 2013). The stress-integrative and responsive brain regions, the hypothalamus, amygdala and hippocampus, following maternal separation stress at PND10 are examined. Interestingly, growth arrest specific 5 (GAS5) is characterized to occupy the nuclear DNA-binding domain of the glucocorticoid receptor and FK506-binding protein 51 (FKBP51) binds to the glucocorticoid receptor in the cytoplasm resulting in reduced nuclear translocation of the glucocorticoid-glucocorticoid receptor complex (Davies et al., 2002; Petta et al., 2016). Consequently, GAS5 and FKBP51 may act to reduce glucocorticoid receptor signaling. Conversely, FKBP52 acts to promote glucocorticoid receptor high affinity for glucocorticoids, thus enhancing glucocorticoid nuclear translocation of the glucocorticoid receptor complex (Schiene-Fischer and Yu, 2001). Thus, we speculate that PAE reduces glucocorticoid receptor function and ultimately signaling through the dysregulated increase in GAS5, FKBP51, and FKBP52 in limbic regions critical for stress regulation. We hypothesized that PAE acts to sensitize stress neuropeptides, endogenous glucocorticoid receptor signaling regulators and TLR4- related and -independent proinflammatory factors in the hippocampus, amygdala and hypothalamus in response to an acute stressor during early postnatal life.

## Materials and methods

### Animals

All procedures adhered to the ethical guidelines for laboratory animals in research (Council, N.R, 2011), which required approval by the Institutional Animal Care and Use Committee (IACUC) of the University of New Mexico Health Sciences Center, and are reported according to ARRIVE Guidelines (Kilkenny et al., 2010). All mice were housed in a temperature (22°C) and light-controlled (reverse light/dark; lights on at 20:00 h) rooms, with standard rodent chow and water available *ad libitum* prior to and during experimentation. Male and female 60-day-old mice (C57BL/6 J; Jackson laboratories, Bar Harbor, ME) were group-housed and acclimated for 1 week prior to single-housing females 1 week prior to initiating the alcohol paradigm. Male mice were grouped two per cage approximately, 1 week prior to initiating breeding.

### Ethanol exposure model

Thirty-two female mice were allowed limited access to ethanol identically as previously described (Brady et al., 2012). Briefly, female mice were randomly assigned to saccharin sweetened water (SAC; saccharin was obtained from Sigma-Aldrich, St. Louis, MO), used as the control, or a SAC-sweetened ethanol solution (ethanol was obtained from Deacon Labs, King of Prussia, PA). Two hours into the dark cycle (10:00), mice were provided access to a solution of either 10% (*w*/*v*) ethanol and 0.066% (*w*/*v*) saccharin or 0.066% (*w*/*v*) saccharin (control) offered in replacement of standard water for a period of 4 h and replaced by water thereafter. On days 1–2, the ethanol concentration (*w*/*v*) was 0%, and increased to 5% on days 3–4 and further increased to 10% on days 5–6. Mice continued to be offered either SAC or 10% ethanol for a 4-h period for ~ 1 week prior to initiating the breeding period. After establishing consistent drinking, a 5-day breeding period commenced, whereby immediately following the 4-h drinking period, females were placed with 2 males and at 08:00 and returned to their individual cages. Access to SAC or 10% ethanol was available throughout gestation. Females were weighed every 3–4 days to confirm pregnancy by weight gain. Following parturition, solutions were decreased to 0% in a stepwise fashion over a period of 6 days. The currently used PAE model has been fully characterized previously (Brady et al., 2012).

### Maternal separation stress

On PND10, pups were subjected to a 4-h maternal separation stress. For the majority of litters (30 of 32), whole litters were randomly assigned to control or maternal separation. All pups from the litter remained in the same cage that was placed in a separate room for 4 h starting at 10:00. Control mice stayed in the home cage with their dams, undisturbed, for 4 h.

### Tissue collection

Immediately following stress or control conditions, pups were sexed by visual examination of external genitalia and then euthanized by rapid decapitation followed by brain removal and dissection on ice of the hypothalamus (HYP), bilateral hippocampus (HPC), and amygdala (AMG) that were placed into homogenization DNase/RNase/Protease-free 1.5 mL disposable pellet mixer microtubes (VWR International; Cat#47747-358), briefly spun down, and immediately snap frozen in liquid nitrogen. All samples were stored at − 80 °C until the time of assay. Tissue samples were selected from different litters to achieve up to an *N* = 10 / study group for each sex. The experimental conditions for males and females were as follows: PAE unstressed, SAC unstressed, PAE stressed, SAC stressed. Final numbers for each group were between 4 and 10.

### RNA extraction and RT-qPCR assay

RNA extraction and RT-qPCR procedures were conducted similarly to that previously described (Noor et al., 2019). Briefly, frozen tissues were retrieved on dry ice and scraped into homogenization tubes or onto a petri dish on dry ice for cutting, ensuring that tissues remained frozen. Total RNA was extracted using the miRNeasy micro kit (Qiagen, catalog # 217084). The petri dish, cutting blade, and forceps were thoroughly cleaned with ethanol, RNAse zap, and DNAse away (Thermo Scientific, MA, United States). The entire tissue sample from the HYP and AMG was processed. A randomly selected half of the HPC within each experimental condition was processed for RNA extraction. Qiazol (200 μl) was added to homogenization tubes containing the frozen tissue, homogenized on ice for 90 s followed by further addition of Qiazol (500 μl), vortexed for 30 s, and incubated at RT for 7-min, which allows dissociation of nucleoprotein complexes, and finally adding 140 μL of chloroform (Sigma-Aldrich; Cat#C2432). The samples were then mixed vigorously for 15 s, incubated for 4 min at RT, mixed vigorously for 10 s, and then centrifuged at 4°C at 12000 × *g* for 15 min. Approximately 300 μl of the aqueous layer was retrieved from the samples and the final elution of RNA was in 20 μl of RNAse/DNase free sterile water. RNA concentration was assessed using NanoDrop (Thermo Scientific, MA, United States). The remaining sample (~300ul) was aliquoted into homogenization DNase/RNase/Protease-free 1.5 mL disposable pellet mixer microtubes (VWR International; Cat#47747-358), briefly spun down and frozen at − 80 °C until protein assay from the AMG.

### RT-qPCR assay

The extracted RNA was diluted to a standardized concentration of 200 ng/ μl (+/− 10 ng/ μl) for all tissues except male hypothalamus. The male hypothalamus was standardized to a concentration of 100 ng/ μl (+/− 5 ng/ μl). The reduced concentration relative to all of the other tissues was due to proportion of samples having been previously processed with compromised sample integrity and consequently, reduced sample material. Half of the male hypothalamus RNA samples had been extracted separately with these resultant lower levels of RNA. For the current experiment, we chose a concentration of 100 ng/μl to ensure adequate sample material collected from each pup. For the cDNA synthesis reaction, the RNA concentration for the reaction was 2.4 μg for all tissues except for male hypothalamus, which was 1.1 ug. cDNA synthesis was performed using the SuperScript^™^ IV VILO^™^ cDNA Synthesis Kit (Invitrogen; Cat#:11754250) per manufacturer’s instructions.

mRNA expression levels were measured and analyzed as previously described (2) with the following modifications. Dilution factors were determined to allow for C_T_ values between 17 and 33, as estimated by prior publications from our lab (Noor et al., 2019) based on their estimated abundance under non-pathological conditions. The housekeeping gene used as an internal control for each reaction was 18 s, and the cDNA required a dilution factor of 1:1,000 for all tissues due to its high abundance in cells. However, for all of the target genes of interest, the following cDNA dilution factors were adjusted down, ranging from 50 to 400-fold dilutions, to account for their relative reduced abundance in cells (Noor et al., 2019). For the amygdala and hippocampus for both males and females, a dilution of 1:8 was used for Gas5, FKBP5, FKBP4, GFAP, and HMGB1, and a dilution of 1:4 was used for TLR4, NLRP3, IκBα, IL-1β, TNF-α, CCL2, IL-10, IL-1R, CRH, AVP, and Tmem119. For female hypothalamus, a dilution of 1:4 was used for targets Gas5, FKBP5, FKBP4, GFAP, HMGB1, TLR4, NLRP3, IL-1β, TNF-α, CCL2, IL-10 and Tmem119. A 1:8 dilution was used CRH, AVP, IL-1R, and IκBα. For male hypothalamus, a dilution of 1:2.5 was used for NLRP3, IL-1β, TNF-α, CCL2, and IL-10, a dilution of 1:5 was used for IκBα. IL-1R, CRH, and AVP, a dilution of 1:10 was used for TLR4 and Tmem119, and a dilution of 1:20 was used for Gas5, FKBP5, FKBP4, GFAP, and HMGB1. All targets were run in triplicate and data are presented as 2^CTnorm/2^CTtarget as previously detailed (Noor et al., 2019).

For the amygdala and hippocampus, all male and female samples were run on the same plate for both the cDNA synthesis reaction and each PCR reaction for each target.

### RT-qPCR validation of results for hypothalamus

A significant majority of the samples from male and female hypothalamus were conducted with standardized RNA inputs for the cDNA reactions *within* male and female groups, but different *between* males (100 ng/μl) and females (200 ng/μl). Additionally, the PCR assays for females were run on separate assay plates from the male samples. Therefore, to ensure that potential sex differences were not due to different RNA concentration (males 100 ng/μl) and on separate plates at the time of assay, we conducted a validation of qRT-PCR reflecting target mRNA levels of males and females using a subset of samples from each experimental group and examined a subset of gene targets, *with cDNA diluted identically*, and males and females assayed together on the same qPCR plate for each target. This separate, smaller experiment using a subset of samples from the hypothalamus was composed of all experimental groups (*n* = 4/group) *with cDNA diluted identically*, at 100 ng/μl for males and females, and assayed together for the following subset of the gene targets: CRH, GAS5, GFAP, NFKBIA and IL1B. All other tissues were assayed using identical standardized RNA inputs for the cDNA reactions as well as run on the same assay plates.

### Protein extraction and analysis

Frozen pellets of amygdalae samples (~300 μl), which were derived from the samples created at the time of total RNA extraction (see above), were thawed on ice and treated with 100% ethanol, 0.3 M of guanidium hydrochloride to recover proteins from a denaturing environment (prior Qiazol-chloroform). Pellets were homogenized, sonicated, and incubated in BSA solution and cell lysis buffer 2 (R&D Systems; Cat# 895347) for purified protein isolation. The supernatant containing the proteins were transferred to final tubes and stored at − 20°C until further assay using the *RC–DC Protein Assay* which is reducing-agent and detergent-agent compatible (Bio-Rad Cat#500-0122). All procedures were performed according the manufacturer’s instructions for RC/DC Protein assay. Briefly, a six-point standard curve in the range 1.25–500 ng/μl was performed using the BSA solution and cell lysis buffer 2 (R&D Systems; Cat# 895347). Samples were diluted 1:15 in cell lysis buffer 2. Standards and samples were added to RC Reagent I (BioRad; Cat# 500-0117) and RC Reagent II (BioRad; Cat# 500-0118) and centrifuged at 12,758 rpm for 5 min, followed by discarding the supernatant. RC Reagent A’, which was prepared by combining Reagent A (BioRad; Cat#500-0113) with Reagent S (BioRad; Cat # 500-0115), was added to the pellet and vortexed. The mixture was then added to DC Reagent B (BioRad; Cat# 500-0114), vortexed and incubated in the dark at RT for 15 min. Triplicate standards and samples (200μl/well) were prepared. Absorbance was measured at 750 nm in Biotek-Syngery-Neo2 plate reader.

### Enzyme-linked immunosorbent assays (ELISA)

The protein expression of CRH, IL-1β, FKBP5 and NLRP3, in mouse amygdala was detected using enzyme-linked immunosorbent assay (ELISA) kits (CRH: Cat#MBS2020732; FKBP5: Cat#MBS2705556; NLRP3: Cat#MBS920134 MyBiosource, United States; IL-1β Cat#MHSLB00 R&D Systems, United States). The relative concentrations for IL-1β and NLRP3 were acquired according to the standard curve generated by microplate reading at 450 nm; wavelength corrections at 540 nm and 570 nm. The relative concentrations for CRH and FKBP5 were acquired according to the standard curve generated by microplate reading at 450 nm.

### Statistical analysis

All statistical analyses were conducted using GraphPad Prism Version 8.4.3 (GraphPad Software Inc.; RRID: SCR002798) as previously described (Noor et al., 2019). Briefly, results were graphed as normalized values (2^Ct normalizer^/2^Ct target^) and analyzed using a 3-way ANOVA to identify main effects and interactions of sex, stress, and/or prenatal alcohol exposure in AMG and HPC. Importantly, prior work established that results from qRT-PCR can be utilized to compare mRNA expression levels from different groups when identical RNA amounts are used for the cDNA synthesis reaction (Ståhlberg et al., 2004).

For the validation experiment of hypothalamus tissue, a 3-way ANOVA was conducted to determine if mRNA levels of chosen target genes between males and females were significantly different when utilizing identical RNA concentration inputs. Similar to that for the validation experiment, mRNA derived from the AMG and the HPC were analyzed using 3-way ANOVA procedures to make direct comparisons between males and females of all target genes. Two-way ANOVA procedures were applied to identify within-sex effects of stress and/or prenatal alcohol exposure in HYP, AMG, and HPC. The results were graphed as fold change using the 2^–ΔΔCT^ method (Livak and Schmittgen, 2001). All graphs represent mean +/− standard error of the mean. The Fisher’s LSD test was used for *post hoc* analysis of between-group differences as determined *a priori.* Within-group outliers were excluded from all analyses as determined by the Grubb’s test (Grubbs, 1969; Iglewicz and Hoaglin, 1993), and applied to the data using the GraphPad online outlier calculator^1^ with *α* = 0.05. Grubb’s test could not be applied to data derived from the HYP validation experiments due to low sample sizes.

## Results

### Validation of mRNA levels between sex in the hypothalamus: comparison of levels when RNA extracted material is utilized at different concentrations (100 vs. 200  ng)

Only tissue from the hypothalamus (HYP) for males and females was assayed differently relative to the amygdala (AMG) and hippocampus (HPC). Specifically, male and female hypothalamic tissue were not assayed on the same plate at the same time, and RNA concentrations between males and females were not the same, which can lead to potential spurious effects caused by differences in PCR amplification efficiencies. Such artificial results can include differences in male relative to female mRNA expression levels. To address this possibility, a validation of qRT-PCR reflecting selected target mRNAs from each stress and neuroimmune category was conducted. The data demonstrate significant sex differences were identified, with significantly greater mRNA expression in female hypothalamus for NFKBIA (*F*_1,15_ = 8.750, *p* = 0.0098), and IL1B (*F*_1,15_ = 5.948, *p* = 0.0276), mRNA (Table 1). A strong trend toward an interaction between sex and PAE in elevated IL1B in was revealed, albeit lacking statistical significance (*F*_1,15_ = 5.730, *p* = 0.0741). No sex differences were detected in mRNA levels for CRH, GAS5 or GFAP (Table 1).

**Table 1:** Three-way ANOVA: validation of methods from a subset of samples from the hypothalamus with cDNA identically diluted to detect sex differences.

	CRH	GAS5	GFAP	NFKBIA	IL1B
Significance	ns	ns	ns	^**^	^*^
Sex with higher expression	**–**	**–**	**–**	F	F

**Figure 1:**
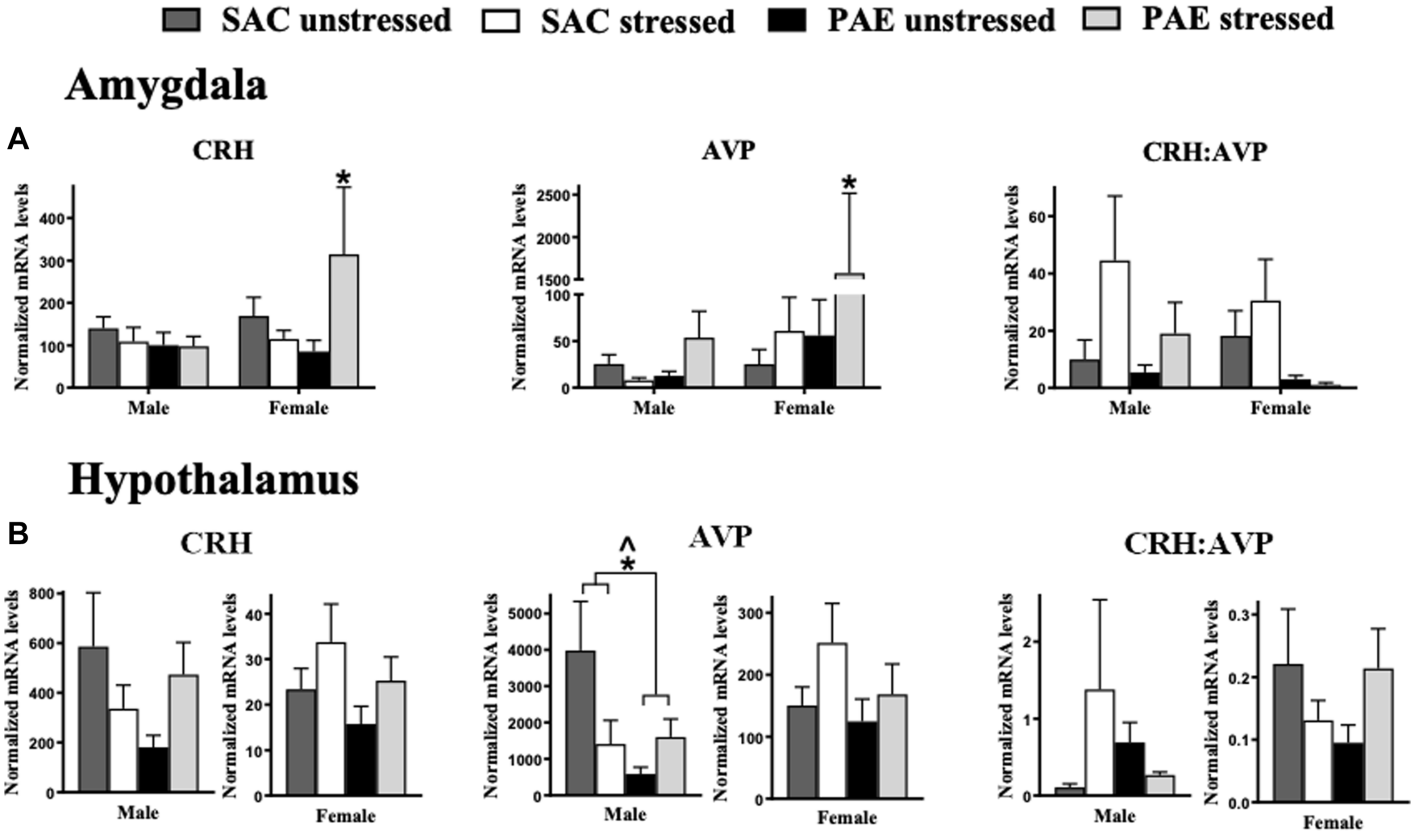
The influence of prenatal alcohol exposure (PAE) and stress exposure on corticotropin releasing hormone (CRH) and arginine vasopressin (AVP) mRNA in the amygdala (AMG) and hypothalamus (HYP). **(A)** In the male amygdala, CRH, AVP, and the ratio of CRH:AVP mRNA levels were not altered by PAE or stress. In females, a significant interaction between PAE and stress results in increased CRH and AVP mRNA levels; ^*^Indicates an interaction between PAE and stress. The ratio of CRH to AVP mRNA are not altered by PAE and/or stress in females. **(B)** In the hypothalamus, CRH mRNA levels and the ratio of CRH:AVP mRNA levels are not altered by PAE or stress in either sex. A main effect of PAE reduces AVP mRNA levels in males. Additionally in males, an interaction of PAE and stress is revealed with lower AVP levels in PAE relative to SAC mice, with disparate responses to stress. No reliable differences are observed in AVP mRNA levels from PAE or stress in female HYP. All results reflect 2-way ANOVA main effects and interactions. *Interaction of PAE and stress = *p* ≤ 0.05. ^main effect of PAE = *p* ≤ 0.05.

**Figure 2:**
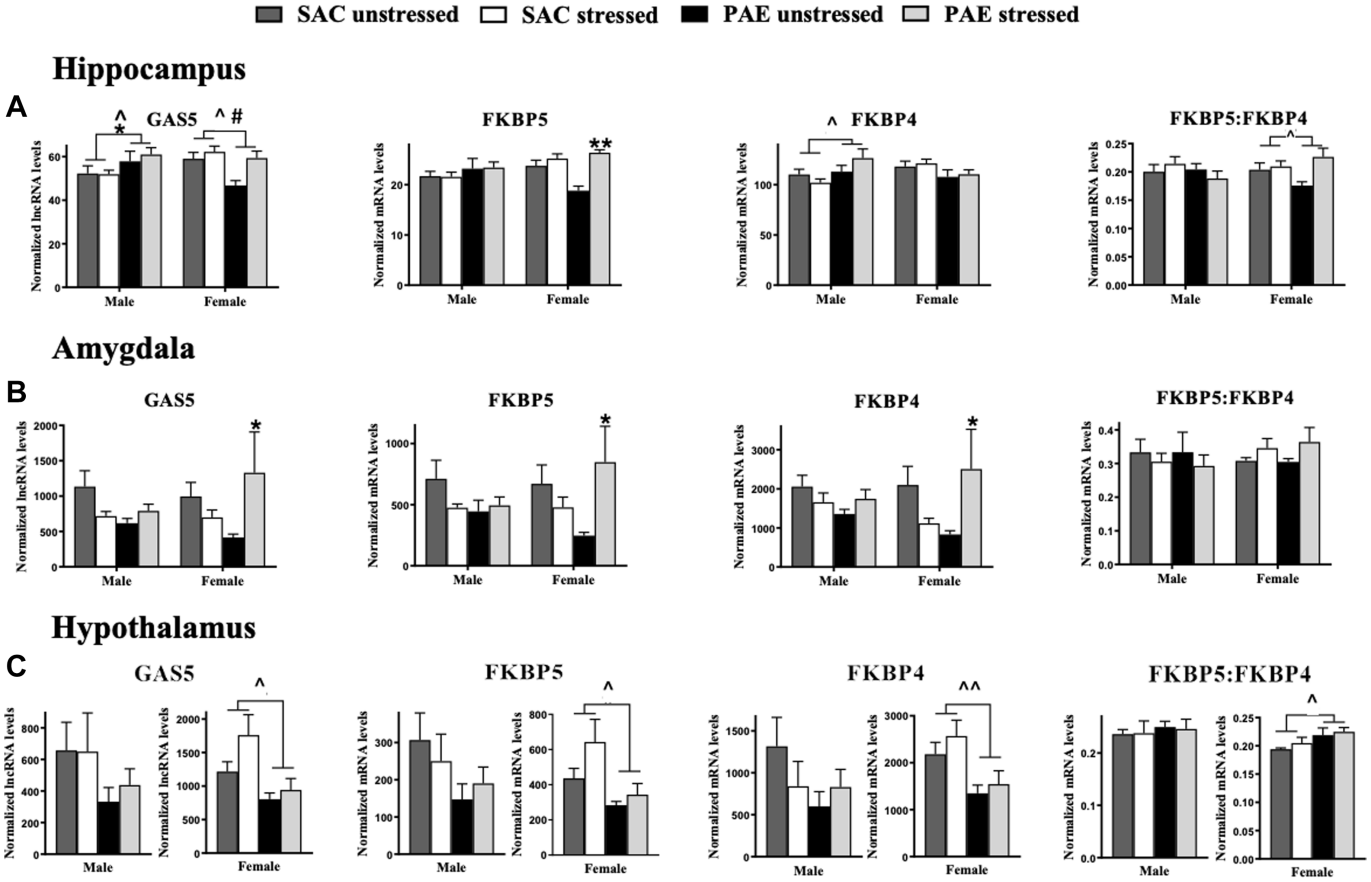
The mRNA of glucocorticoid receptor signaling regulators are altered by PAE and stress in the hippocampus amygdala and hypothalamus **(A)** Hippocampus (HPC): a main effect of PAE results in increased GAS5 lncRNA levels in male mice and decreased GAS5 lncRNA levels in female mice, as well as a main effect of stress that increases Gas5 expression in females. In males, FKBP5 mRNA levels are not altered by PAE or stress, but in females with PAE, a main effect of stress increases FKBP5 expression. In males, a main effect of PAE increases FKBP4 expression. Neither PAE nor stress do not alter FKBP4 mRNA levels in females. The ratio of FKBP5 to FKBP4 mRNA levels were not affected by PAE or stress in males. In females, a main effect of PAE reveals increases in FKBP5: FKBP4. **(B)** Amygdala (AMG): In the male amygdala, GAS5, FKBP5, FKBP4, and FKBP5:FKBP4 ratio mRNA levels were not influenced by PAE or stress. In females with PAE, an interaction with stress increases expression of GAS5, FKBP5, and FKBP4. Stress and/or PAE does not alter FKBP5:FKBP4 mRNA in females. **(C)** Hypothalamus (HYP): For the male hypothalamus, GAS5, FKBP5, FKBP4, and the ratio of FKBP5 to FKBP4 mRNA levels are not influenced by PAE or stress. However, a main effect of PAE in females results in blunted levels of GAS5, FKBP5, and FKBP4 mRNA independent of stress exposure, and FKBP5:FKBP4 mRNA is increased by PAE. GAS5 lncRNA: growth arrest specific 5 long non-coding RNA; FKBP5: FK506-binding protein 51; FKBP4: FK506-binding protein 52. All results reflect 2-way ANOVA main effects of PAE and stress and interactions of PAE and stress. ^*^Interaction = *p* ≤ 0.05, interaction ^**^ = *p* ≤ 0.01. ^main effect of PAE = *p* < 0.05, ^^main effect of PAE = *p* < 0.005, ^#^main effect of stress = *p* < 0.05.

**Figure 3:**
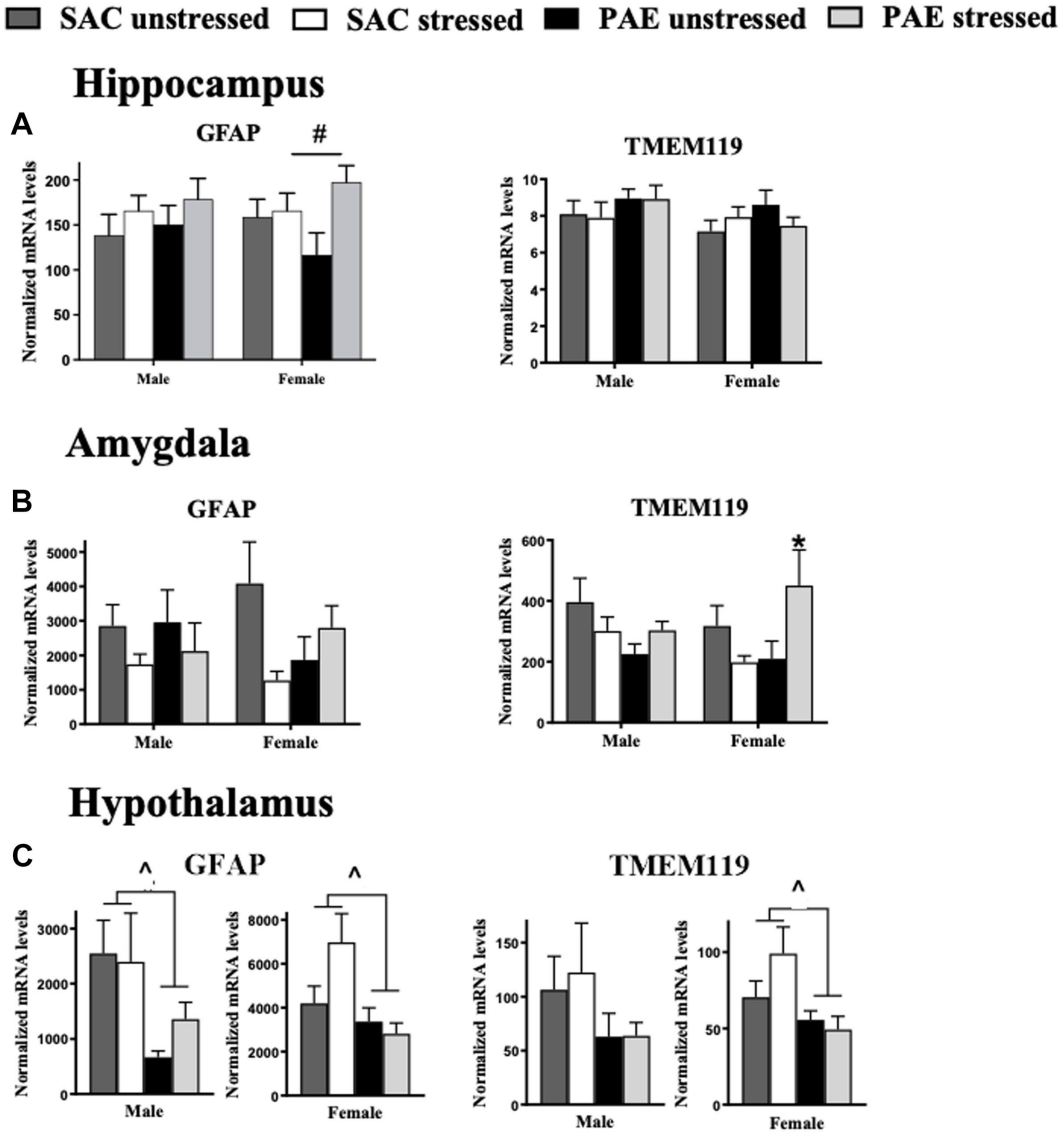
Glial activation markers for astrocytes and microglia mRNA expression in the hippocampus, amygdala, and hypothalamus. **(A)** Hippocampus (HPC): male GFAP and TMEM119 mRNA levels are not altered by prenatal alcohol exposure (PAE) or stress. In females with PAE, an interaction with stress increases GFAP expression, while no changes in TMEM119 expression between groups are observed. **(B)** Amygdala (AMG): For the amygdala, male and female GFAP mRNA levels are not altered as a result of PAE or stress. In males, TMEM119 expression is similarly unaffected by PAE or stress. However, females with PAE revealed a significant interaction with stress leading to elevated TMEM119 mRNA levels. **(C)** Hypothalamus (HYP): For the hypothalamus, a main effect of PAE induced blunted GFAP mRNA levels in both male and females. While no significant changes in TMEM119 expression between groups are observed in males, a main effect of PAE blunted TMEM119 expression observed in females independent of stress exposure. Astrocytes, glial fibrillary acid protein (GFAP); microglia, transmembrane protein 119 (TMEM119). All results reflect 2-way ANOVA main effects and interactions. ^#^main effect of stress = *p* < 0.05, ^main effect of PAE = *p* < 0.05, ^*^Interaction of PAE and stress = *p* < 0.05.

**Figure 4:**
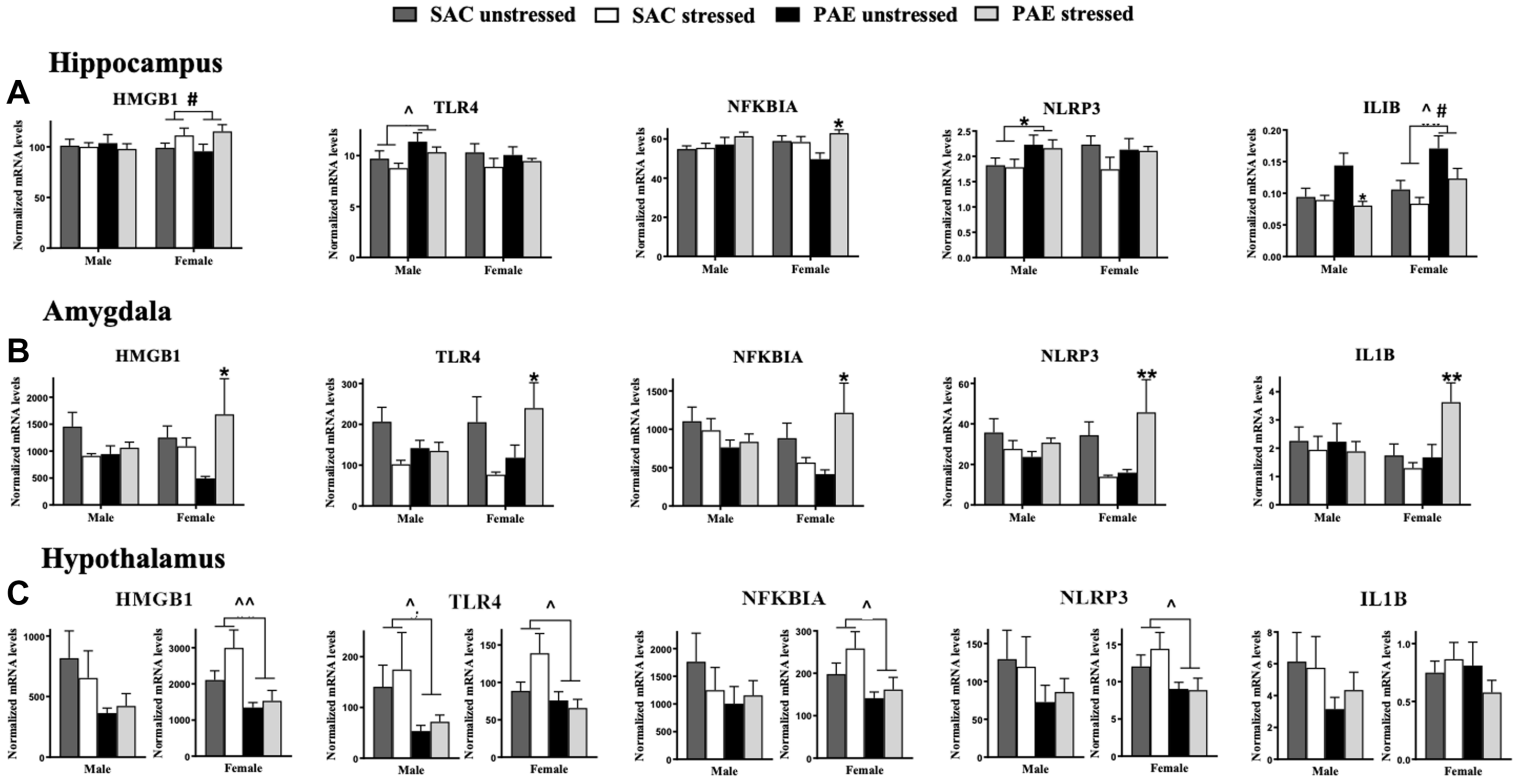
mRNA alterations in factors related to the TLR4 signaling cascade HMGB1, TLR4, NFKBIA, NLRP3, and ILIB. **(A)** Hippocampus (HPC): HMGB1 mRNA levels are not altered by prenatal alcohol exposure (PAE) or stress in males. A main effect of stress in females results in elevated HMGB1 expression. A main effect of PAE results in increased TLR4 expression, while no group differences are observed in TLR4 expression levels in females. While no changes are observed in male NFKBIA, females with PAE reveal an interaction with stress that leads to increases NFKBIA mRNA levels. Male offspring reveal a main effect of PAE that increases NLRP3 expression, with no meaningful differences observed between groups in females. In females, main effects of stress and PAE result in increased IL1B mRNA levels while in males, an interaction between PAE and stress result in decreased IL1B mRNA levels relative to SAC male mice. **(B)** Amygdala (AMG): In females, a significant interaction of PAE and stress increases expression of HMGB1, TLR4, NFKBIA, NLRP3, and IL1B while no alterations in mRNAs from SAC female mice were observed. No reliable mRNA changes were observed in male amygdala. **(C)** Hypothalamus (HYP): In males, levels of HMGB1 are not significantly different between treatment groups, while blunted HMGB1 levels are observed in females due to a main effect of PAE independent of stress exposure. Similarly, TLR4 expression is significantly blunted due to a main effect of PAE males and females. Additionally in females, but not males, PAE blunted NFKBIA, and NLRP3 mRNA levels independent of stress exposure. HMGB1, high mobility group box 1; TLR4, toll-like receptor 4; NFKBIA, NF-κB inhibitor alpha; NLRP3, NOD-, LRR- and pyrin domain-containing protein 3; ILIB, interleukin-1 beta. All results reflect 2-way ANOVA main effects and interactions. ^#^main effect of stress = *p* < 0.05, ^main effect of PAE = *p* < 0.05, ^^main effect of PAE = *p* < 0.01, ^*^Interaction of PAE and stress = *p* < 0.05, ^**^ = *p* < 0.01.

**Figure 5:**
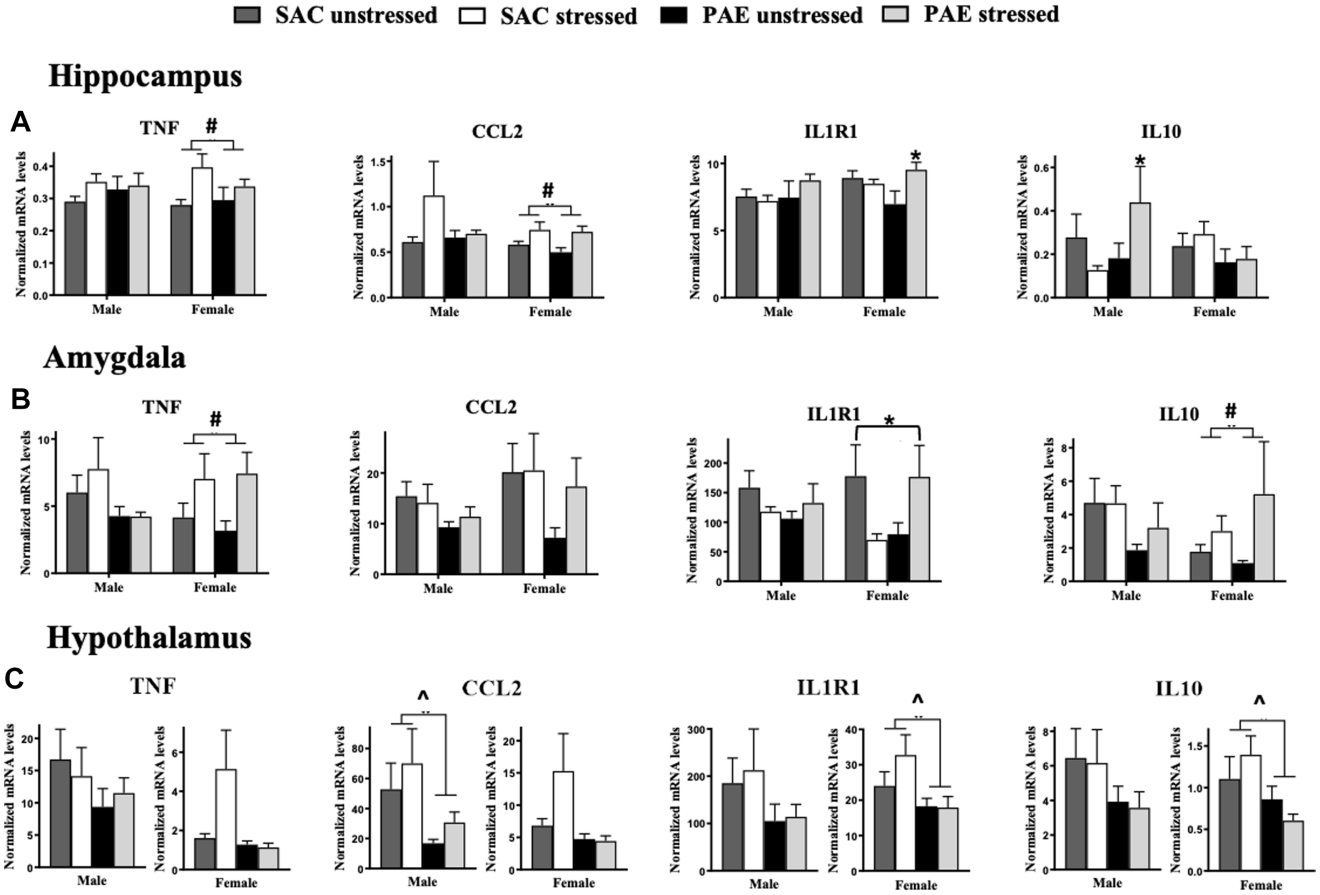
Alterations in mRNA expression of TNF, CCL2, IL1R1, and IL10. **(A)** Hippocampus (HPC): In females, a main effect of stress increases TNF and CCL2 mRNA levels, while no changes between treatment groups are observed in males. A significant interaction of PAE and stress results in elevated IL1R1 mRNA expression levels in females, while no changes between treatment groups are observed in males. Levels of IL10 mRNA expression are significantly elevated in males as a result of a PAE and stress interaction, while no changes in IL-10 levels are observed between treatment groups in females. **(B)** Amygdala (AMG): In females, but not males, a main effect of stress results in increased TNF and IL-10 mRNA levels, independent of PAE. An interaction of PAE and stress differentially alters in IL1R1 expression in females. Levels of CCL2 mRNA are not significantly different between treatment groups in males and females. While no meaningful differences are observed between groups in males **(C)** Hypothalamus (HYP): Despite strong trends for diminished TNF and CCL2 expression levels in females, mRNA levels were not significantly influenced by PAE, while PAE significantly reduced CCL2 in males. Additionally, a main effect of PAE consistently blunted IL1R1 and IL10 mRNA levels in females, with strong trends of blunted IL1R1 and IL-10 mRNA levels in males with PAE. TNF, tumor necrosis factor alpha; CCL2, chemokine ligand 2; IL1R1, interleukin-1 receptor 1; IL10, interleukin-10. All results reflect 2-way ANOVA main effects and interactions. ^main effect of PAE = *p* < 0.05, ^#^main effect of stress = *p* < 0.05, *main interaction of stress and PAE = *p* ≤ 0.

A significant majority of the samples from male and female hypothalamus were conducted with standardized RNA inputs for the cDNA reactions within male and female groups, but different between males (100 ng/μl) and females (200 ng/μl). To address potential sex differences due to different RNA concentrations, a separate and smaller experiment was performed with a subset of samples from the hypothalamus from each experimental group (*n* = 4/group) with cDNA diluted identically; that is at 100 ng/ul for males and females, which were assayed together for a subset of gene targets. No sex differences were detected in mRNA levels for CRH, GAS5 or GFAP, but females demonstrated significantly greater mRNA expression for NFKBIA and IL1B. These results support that the pattern of sex differences observed for hypothalamic CRH, GAS5, GFAP, NFKBIA and IL1B mRNA levels from the much larger study (Figures 1–5; *n* = 10) reflect true mRNA levels of despite applying different RNA concentrations between male and female samples for assay. CRH, corticotropin releasing hormone; GAS5, growth arrest specific 5; GFAP, glial fibrillary acid protein; NFKBIA, NF-κB inhibitor alpha; IL1B, interleukin-1 beta. ^*^ = *p* ≤ 0.05, ^**^ = *p* ≤ 0.01.

These data show a similar pattern of sex comparability observed for hypothalamic CRH, GAS5, GFAP, NFKBIA and IL1B mRNA levels relative to those levels observed from the much larger study reflected in Figures 1B, 2C–5C, despite lower RNA concentrations between male and female samples for the hypothalamic assay. Hence, while results of mRNA levels from the larger study between males and females were either on similar y-axis scales, or were 2–10 -fold different, or 40-fold different with CRH (Figures 1B, 2C, 3C, 4C, 5C), the *pattern* of sex differences remained very similar relative to the smaller validation study represented in Table 1. The selected mRNA factors from the smaller validation study represent 1–2 targets from each of the following categories: the stress peptide, CRH; the glucocorticoid receptor signaling regulator, GAS5; a glial activation marker, astrocyte GFAP; the TLR4-related activation factors, NFKBIA and IL1B, as represented in Table 1. Thus, no additional analyses for main effects of sex on other targets were conducted for hypothalamus.

### Characterization of stress-associated mRNA levels of CRH and AVP

#### Sex differences in the amygdala

Clear sex differences in mRNA levels for AVP from the amygdala were observed, with significantly greater levels in females than males (F_1,36_ = 5.730, *p* = 0.022), with significant increases observed from PAE and following stress (F_1,36_ = 4.445, *p* = 0.042; Figure 1A; Table 2). It is notable that a strong trend toward elevated CRH mRNA levels in females was observed (F_1,39_ = 2.849, *p* = 0.099), with notable increases from PAE and following stress (F_1,39_ = 3.266, *p* = 0.078). No sex differences were identified in the CRH:AVP ratio. Additionally, both PAE and stress robustly increased mRNA CRH (F_1,39_ = 4.901, *p* = 0.033) and AVP (F_1,36_ = 5.206, *p* = 0.029; Figure 1A; Table 2). The robust changes in mRNA levels as a consequence of both PAE and stress prompted additional analyses for potential within sex differences, as noted below and in Table 3.

**Table 2:** Summary of 3-way ANOVA: hippocampus and amygdala for effects of sex, prenatal exposure and stress.

	CRH	AVP	CRH: AVP	GAS5	FKBP5	FKBP4	FKBP5: FKBP4	GFAP	TMEM119	HMGB1	TLR4	IκBα	NLRP3	IL-1β	TNF-α	CCL2	IL-1R	IL-10
Main effect of sex																		
Hippocampus																		
Amygdala		*																
Main effect of prenatal exposure																		
Hippocampus						**							*	**				
Amygdala		*																
Main effect of stress exposure																		
Hippocampus				*	**			*				*		**	*			
Amygdala		*																
Main interaction between sex and prenatal exposure																		
Hippocampus				**	*	**												
Amygdala		*																
Main interaction between sex and stress exposure																		
Hippocampus					**					*								
Amygdala		*								*								
Main interaction between prenatal exposure and stress exposure																		
Hippocampus					*							*					*	
Amygdala	*	*		**	**	**			**	**	**	*	***				**	
Main interaction between sex, prenatal exposure, and stress exposure																		
Hippocampus																		*
Amygdala		*											*					

The table represents a summary of 3-way ANOVA analyses procedures of all targets examined in the hippocampus and the amygdala for (1) main effects of sex, (2) prenatal exposure and (3) Stress and interactions between (4) sex and prenatal exposure, (5) sex and stress exposure, (6) prenatal exposure and stress exposure, and (7) sex, prenatal exposure and stress exposure. CRH, corticotropin releasing hormone; AVP, arginine vasopressin; GAS5, growth arrest specific 5; FKBP5, FK506-binding protein 51; FKBP4, FK506-binding protein 52; GFAP, glial fibrillary acid protein; TMEM119, transmembrane protein 119; HMGB1, high mobility group box 1; TLR4, toll-like receptor 4; NFKBIA, NF-κB inhibitor alpha; NLRP3, NOD-like receptor family pyrin domain-containing protein 3; IL1B, interleukin-1 beta; TNF, tumor necrosis factor alpha; CCL2, C-C motif chemokine ligand 2; IL1R1, interleukin-1 receptor 1; IL10, interleukin-10. All main effects and interactions are indicated by ^*^, ^*^ = *p* ≤ 0.05, ^**^ = *p* ≤ 0.01, ^***^ = *p* ≤ 0.001.

**Table 3:** Two-way ANOVA: hippocampus, amygdala, and hypothalamus for prenatal exposure and stress.

		CRH	AVP	CRH: AVP	GAS5	FKBP51	FKBP52	FKBP51: FKBP52	GFAP	TMEM119	HMGB1	TLR4	IκBα	NLRP3	IL-1β	TNF-α	CCL2	IL-1R	IL-10
Within-sex main interaction between prenatal and stress exposure																			
Male	Hipp														*				*
	Amygdala																		
	Hypothal		*																
Fem	Hipp					**							*					*	
	Amygdala	*	*		*	*	*			*	*	*	*	**	**			*	
	Hypothal																		
Within-sex main effect of prenatal exposure																			
Male	Hipp				*		*							*					
	Amygdala																		
	Hypothal		*						*			*					*		
Fem	Hipp				*										**				
	Amygdala		*												*				
	Hypothal				*	*	**	*	*	*	**	*	*	*				*	*
Within-sex main effect of stress exposure																			
Male	Hipp											*			*				
	Amygdala																		
	Hypothal																		
Fem	Hipp				*	****		*	*		*		*		*	*	**		
	Amygdala		*													*			*
	Hypothal																		

The table represents a summary of within-sex analyses using 2-way ANOVA procedures of all targets examined in the hippocampus, amygdala, and the hypothalamus. For all tissue regions, main effects of (1) prenatal exposure and (2) stress exposure, and an interaction between prenatal exposure and stress exposure are reported. CRH, corticotropin releasing hormone; AVP, arginine vasopressin; GAS5, growth arrest specific 5; FKBP5, FK506-binding protein 51; FKBP4, FK506-binding protein 52; GFAP, glial fibrillary acid protein; TMEM119, transmembrane protein 119; HMGB1, high mobility group box 1; TLR4, toll-like receptor 4; NFKBIA, NF-κB inhibitor alpha; NLRP3, NOD-like receptor family pyrin domain-containing protein 3; IL1B, interleukin-1 beta; TNF, tumor necrosis factor alpha; CCL2, C-C motif chemokine ligand 2; IL1R1, interleukin-1 receptor 1; IL10, interleukin-10. All main effects and interactions are indicated by ^*^, ^*^ = *p* ≤ 0.05, ^**^ = *p* ≤ 0.01, ^***^ = *p* ≤ 0.001, ^****^ = *p* ≤ 0.0001.

#### Impact of PAE and/or stress in the amygdala

Two-way ANOVAs were conducted to address potential PAE and/or stress induced gene expression (Table 3) within males and females. PAE, stress or the interaction of PAE and stress on target gene expression are represented in Figure 1A, and demonstrate that CRH and AVP expression within male AMG were not altered by PAE or stress. However, in females with PAE and following stress, CRH (F_1,19_ = 4.717, *p* = 0.043) and AVP (F_1,18_ = 4.827, *p* = 0.041) mRNA levels were increased in female AMG. Robust impact of PAE (F_1,18_ = 5.237, *p* = 0.034) and stress (F _1,18_ = 5.305, *p* = 0.033) on AVP mRNA levels were observed. The ratio of CRH to AVP mRNA levels revealed no effects of PAE or stress within male or female AMG (Figure 1A; Table 3).

#### Impact of PAE and/or stress in the hypothalamus

CRH mRNA levels were not influenced by PAE or stress in either males or females (Figure 1B; Table 3). However, AVP mRNA levels in males was lower as a consequence of PAE (F _1,19_ = 4.396, *p* = 0.049) as well as an impact of both PAE and stress (F _1,21_ = 5.542, *p* = 0.029). Relative to unstressed conditions, AVP mRNA levels were divergent following stress in PAE and SAC male HYP. That is, stress decreased SAC AVP expression and increased PAE AVP expression. Neither PAE nor stress influenced the expression of AVP in female HYP or of the ratio of CRH to AVP in male or female HYP. Two-way ANOVA within males and females was utilized to determine these main effects of PAE and/or stress on the expression of stress-associated genes within the HYP (Table 3).

### Characterization of regulators of glucocorticoid receptor signaling: levels of lncRNA for GAS5, and mRNA for FKBP5 and FKBP4

#### Sex differences in the hippocampus and amygdala

For the HPC, sex contributed significantly in observed differences in mRNA levels in mice with PAE for GAS5 (F _1,58_ = 10.94, *p* = 0.0016), FKBP5 (F _1,57_ = 5.138, *p* = 0.027), FKBP4 (F _1,57_ = 9.642, *p* = 0.003), as revealed by an interaction between sex and PAE (Figure 2A; Table 2). In general, female HPC reveals greater changes than observed in males.

For the AMG, there were no significant differences between male and female levels of GAS5 lncRNA and FKBP5, FKBP4, and the ratio of FKBP5 to FKBP4 mRNA, as indicated by 3-way ANOVA analyses (Figure 2B; Table 2). However, further analyses were performed within males and females, as detailed below.

#### Impact of PAE and/or stress in the hippocampus

In male HPC, greater levels of GAS5 lncRNA levels were observed in PAE relative to SAC mice (F _1,26_ = 4.741, *p* = 0.039), but in female HPC, lower levels of GAS5 lncRNA levels were observed in PAE relative to SAC mice (F _1,32_ = 6.367, *p* = 0.017; Figure 2A; Table 3). Among females, stress resulted in increased GAS5 lncRNA levels (F _1,32_ = 6.932, *p* = 0.013; Table 3). In male HPC, FKBP5 mRNA levels were not altered by PAE or stress, but female HPC FKBP5 mRNA levels increase in response to stress (F _1,31_ = 21.75, *p* < 0.0001), an effect that was driven by FKBP5 mRNA elevations in PAE but not SAC mice following stress (F _1,31_ = 9.979, *p* = 0.004). Within males, PAE led to higher levels of FKBP4 expression (F _1,26_ = 5.500, *p* = 0.027). Neither PAE nor stress did not influence the expression of FKBP4 mRNA in female HPC or of the ratio of FKBP5 to FKBP4 mRNA in male HPC. The ratio of FKBP5 to FKBP4 mRNA levels increased in response to stress in female HPC (F _1,32_ = 4.951, *p* = 0.033; Figure 2A; Table 3).

#### Impact of PAE and/or stress in the amygdala

We explored whether the gene expression of glucocorticoid receptor signaling regulators were influenced by PAE and/or stress within males or females in the AMG utilizing 2-way ANOVA procedures (Table 3). In the males, GAS5 lncRNA expression levels were not altered by PAE or stress, however, in females, PAE increased GAS5 lncRNA expression following stress that was not observed SAC mice (F _1,20_ = 6.097, *p* = 0.023). Strikingly, stress-driven elevations of FKBP5 and FKBP4 expression only in female mice with PAE was observed (F _1,20_ = 6.545, *p* = 0.019; F _1,19_ = 6.953, *p* = 0.016), while FKBP5 and FKBP4 expression was not reliably altered by PAE or stress in male mice (Figure 2B; Table 3). Additionally, the ratio of FKBP5 to FKBP4 mRNA levels was not altered by PAE or stress within either sex.

#### Impact of PAE and/or stress in the hypothalamus

In females, expression levels of GAS, FKBP5, and FKBP4 were consistently lower in mice with PAE relative to SAC-treated mice (F _1,31_ = 7.169, *p* = 0.012; F _1,31_ = 5.868, *p* = 0.022; F _1,30_ = 9.260, *p* = 0.005, respectively). The ratio of FKBP5 to FKBP4 mRNA levels was additionally increased in female offspring with PAE (F _1,31_ = 6.474, *p* = 0.016; Figure 2C; Table 3). In males, there were no effects of PAE or stress on the expression of GAS5, FKBP5, FKBP4, or the ratio of FKBP5 to FKBP4.

### Characterization of astrocyte and microglial mRNA levels of GFAP and TMEM119, respectively

#### Sex differences in the hippocampus and amygdala

The mRNA expression levels of the astrocyte activation marker, GFAP and the microglial activation marker, TMEM119, did not consistently and significantly differ between males and females in either the HPC or AMG (Figures 3A,B; Table 2).

#### Impact of PAE and/or stress in the hippocampus

While GFAP mRNA levels increased in response to stress in females (F _1,32_ = 4.534, *p* = 0.041), in the males, GFAP mRNA expression was not influenced by either PAE or stress. TMEM119 mRNA levels were not influenced by PAE or stress in either sex, as examined by applying a 2-way ANOVA procedures (Figure 3A; Table 3).

#### Impact of PAE and/or stress in the amygdala

A striking interaction between PAE and stress induced elevated microglial TMEM119 mRNA levels in females (F _1,19_ = 7.273, *p* = 0.014) with no such changes observed in males. Astrocyte GFAP mRNA expression levels were not altered by either PAE or stress in either males or females, as revealed by 2-way ANOVA procedures to explore possible within-sex effects in the AMG (Figure 3B).

#### Impact of PAE and/or stress in the hypothalamus

While differences in GFAP mRNA levels in the HYP were not dependent on sex (Figure 3C), as shown by a 3-way ANOVA for GFAP in the smaller validation experiment (*p* = 0.067; Table 1), PAE on glial activation markers within sex in the HYP revealed robust differences. Astrocyte GFAP mRNA expression in male mice was blunted by PAE (F _1,17_ = 5.525, *p* = 0.031) as well as within females (F _1,30_ = 6.374, *p* = 0.017). Microglial TMEM119 expression was blunted in female mice by PAE (F _1,31_ = 5.687, *p* = 0.023), while TMEM119 mRNA levels were not significantly changed by PAE or stress in males (Figure 3C; Table 3).

### Characterization of TLR4 activation pathway markers: mRNA levels of HMGB1, TLR4, NFKBIA, NLRP3, and IL1B

#### Sex differences in the hippocampus and amygdala

For the HPC, a significant impact of sex and stress lead to alterations in HMGB1, with females demonstrating greater HMGB1 mRNA levels following stress, but independent of PAE (F _1,58_ = 4.341, *p* = 0.042; Figure 4A; Table 2). The mRNA levels of the remaining TLR4-related factors;TLR4, NFKBIA, NLRP3, and IL1B were not impacted by sex, as revealed by 3-way ANOVA analyses procedures (Figure 4A; Table 3).

For the AMG, a significant impact of sex and stress lead to alterations in HMGB1, with females demonstrating greater HMGB1 mRNA levels following stress, but independent of PAE (F _1,40_ = 4.415, *p* = 0.0420; Figure 4B; Table 2). Additionally, sex interacted with PAE and stress, which increased NLRP3 mRNA levels in females, but not males (F _1,38_ = 4.101, *p* = 0.0499; Figure 4B; Table 2).

#### Impact of PAE and/or stress in the hippocampus

The impact of PAE and/or stress on TLR4-associated mRNA expression in the HPC within sex was analyzed using 2-way ANOVA (Figure 4A; Table 3). While the expression of HMGB1 was not altered by PAE or stress in males, HMGB1 mRNA levels were increased by stress treatment in female HPC (F _1,32_ = 5.783, *p* = 0.022). TLR4 expression was increased by PAE in males (F _1,26_ = 5.062, *p* = 0.033), while in females, TLR4 expression remained unaltered by PAE or stress. In males, NFKBIA expression was also not influenced by PAE or stress, while in females, stress increased NFKBIA expression (F _1,32_ = 5.847, *p* = 0.022), an effect that reflected stress-induced elevations of NFKBIA mRNA by PAE but not SAC female mice (F _1,32_ = 6.856, *p* = 0.013). NLRP3 mRNA levels were greater by PAE in males (F _1,26_ = 5.130, *p* = 0.032), while NLRP3 remained unchanged in females. In males, stress-induced decreased IL1B expression (F _1,24_ = 6.055, *p* = 0.021) was observed in mice with PAE, with an interaction between PAE and stress revealed in males (F _1,24_ = 4.453, *p* = 0.046; Figure 4A; Table 3). No changes in IL-1B expression were detected from SAC-exposed offspring under basal or following stressed conditions. In females, PAE increased the overall IL1B expression (F _1,32_ = 11.99, *p* = 0.001), with stress exposure resulting in decreased IL1B mRNA a (F _1,32_ = 5.396, *p* = 0.027; Figure 4A; Table 3).

#### Impact of PAE and/or stress in the amygdala

Alterations in TLR4-associated factors in mRNA expression of the AMG as a consequence of PAE and/or stress were analyzed within sex using 2-way ANOVA (Figure 4B; Table 3). In male offspring, significant differences as a consequence of PAE and stress in HMGB1, TLR4, NFKBIA, and IL1B mRNA expression levels were absent. Conversely, in females, a striking interaction of mice with PAE that were exposed to stress produced a consistent increase in all of the TLR4-related factors measured. That is, increased HMGB1 (F _1,20_ = 5.721, *p* = 0.027), TLR4 (F _1,19_ = 6.848, *p* = 0.017), NFKBIA (F _1,18_ = 8.264, *p* = 0.010), NLRP3 (F _1,18_ = 11.96, *p* = 0.003), and IL1B (F _1,20_ = 8.144, *p* = 0.010) mRNA expression was observed (Figure 4B; Table 3).

#### Impact of PAE and/or stress in the hypothalamus

The impact of PAE and/or stress on TLR4-assoicated inflammatory factors were examined within sex in the HYP by 2-way ANOVA (Figure 4C; Table 3). In males, while mRNA levels for HMGB1, NFKBIA, NLRP3, or IL1B were not impacted by PAE and/or stress, TLR4 mRNA levels were blunted in male by PAE (F _1,23_ = 4.651, *p* = 0.041). However, the mRNA expression pattern analyzed in females was remarkably different. Females with PAE revealed a significant and robustly different pattern, with blunted expression of HMGB1 (F _1,31_ = 8.629, *p* = 0.006), TLR4 (F _1,30_ = 5.005, *p* = 0.033), NFKBIA (F _1,30_ = 5.503, *p* = 0.026), and NLRP3 (F _1,31_ = 5.079, *p* = 0.031) compared to SAC treated females. IL1B expression in the female was not significantly influenced by PAE or stress (Figure 4C; Table 3).

### Characterization of mRNA levels of neuroimmune factors that influence TLR4 actions; proinflammatory cytokine TNF, chemokine CCL2, cytokine receptor IL1R1, and anti-inflammatory cytokine IL10

#### Sex differences in the hippocampus and amygdala

While sex alone did not impact mRNA levels of TNF, CCL2, IL1R1, or IL10 in the HPC or AMG, a significant impact of sex with PAE and stress induced increases in the anti-inflammatory cytokine, IL-10 in male HPC (F _1,56_ = 4.209, *p* = 0.045), as revealed by a 3-way ANOVA (Figure 5A; Table 2). The levels of IL-10 mRNA in females are observed lowest in females with PAE (Figure 5A). No other interactions with sex were observed between male and female mRNA expression levels of TNF, CCL2, or IL1R1, in the HPC or AMG.

#### Impact of PAE and/or stress in the hippocampus

Within sex comparisons were examined to identify the influence of PAE and/or stress on the mRNA expression of pro- and anti-inflammatory factors in the HPC (Figure 5A; Table 3), as analyzed by 2-way ANOVA procedures. While significant increases in IL10 mRNA levels were observed in male offspring with PAE exposed to stress relative to mRNA changes in SAC offspring (F _1,25_ = 4.558, *p* = 0.043), TNF, CCL2, and IL1R1 mRNA levels were not altered by either PAE or stress. However, in females, increases in mRNA levels as a consequence of stress were observed for TNF (F _1,32_ = 6.440, *p* = 0.016), and CCL2 (F _1,30_ = 8.815, *p* = 0.006). A robust interaction was revealed in female mice with PAE exposed to stress as observed by significant increases in IL1R1 expression (F _1,31_ = 6.275, *p* = 0.018; Figure 5A; Table 3).

#### Impact of PAE and/or stress in the amygdala

Within sex examination of the effects of PAE and/or stress on mRNA expression in the AMG applying 2-way ANOVA procedures revealed main effects of stress and an interaction between PAE and stress (Figure 5B; Table 3). Specifically in females, TNF (F _1,20_ = 5.966, *p* = 0.024), and IL-10 (F _1,20_ = 4.875, *p* = 0.039) expression increased as a consequence of stress exposure while no changes in CCL2 and IL1R1 from stress alone were observed. Additionally, no main effects of PAE were revealed for TNF, CCL2, IL1R1, or IL10 mRNA levels. However, in female mice with PAE and exposed to stress, divergent IL1R1 mRNA expression levels were observed (F _1,20_ = 5.761, *p* = 0.026; Figure 5B; Table 3).

#### Impact of PAE and/or stress in the hypothalamus

For within sex, the impact of PAE and/or stress on mRNA expression levels were examined in the HYP of males and females by applying 2-way ANOVAs (Figure 5C; Table 3). Although strong trends of blunted mRNA expression levels were observed in females, TNF and CCL2 mRNA levels were not significantly influenced by PAE or stress in females, while in males, CCL2 mRNA expression was significantly decreased only in PAE mice (F _1,18_ = 4.466, *p* = 0.049). While no IL-1R1 and IL-10 expression level differences were observed in males between groups, females with PAE revealed blunted IL1R1 and IL10 mRNA expression levels independent of stress exposure (F _1,31_ = 4.868, *p* = 0.035; F _1,31_ = 5.465, *p* = 0.026, respectively).

### Characterization of protein in the amygdala for CRH, IL-1b, FKBP5 and NLRP3

For within sex, the impact of PAE and/or stress on protein levels from the amygdala were examined for the stress peptide, CRH, and pro inflammatory *IL-1β and NLRP3, and the glucocorticoid receptor signaling regulator, FKBP5* by applying 2-way ANOVAs (Table 4). Within sex protein analysis from samples that were left over from mRNA extraction yielded low total protein levels. Despite these low yields, either significant differences or strong trends were detected in CRH (*F*_(1,18)_ = 4.617, *p* < 0.05), and IL-1β (*F*_(1,19)_ = 4.132, *p* = 0.05) levels, respectively, from PAE-treated male offspring independent of stress exposure. While it was predicted that protein analyses from the amygdala would reveal group differences that would mirror changes observed in mRNA expression levels, it was not surprising that most of the protein analyses did not result in significant differences between treatment groups, mainly due to the exceptionally low protein yields.

**Table 4:** Protein from amygdala (pg/100 ng).

		CRH							IL-β							FKBP5							NLRP3						
		SAC			PAE				SAC			PAE				SAC			PAE				SAC			PAE			
		AVE	SD	*N*	AVE	SD	*N*	*P*	AVE	SD	*N*	AVE	SD	*N*	*P*	AVE	SD	*N*	AVE	SD	*N*	*p*	AVE	SD	*N*	AVE	SD	*N*	*p*
M	No stress	734.6	539.8	7	1243.5	906.3	6	*	0.9	0.9	7	# 1.5	0.98	6	#	16.6	10.5	5	30.2	26.6	6	NS	51.0	33.7	7	73.7	49.7	6	NS
M	Stress	654.8	202.8	6	1320.1	538	3	*	0.7	0.3	7	# 1.5	0.6	3	#	12.6	7.6	6	17.6	10.2	3	NS	44.7	27.4	7	61.3	1.8	2	NS
																													
F	No stress	1171.2	935.0	9	773.3	209.4	6	NS	0.9	0.9	10	0.7	0.3	6	NS	15.3	14.3	9	12.3	5.9	6	NS	41.2	23.0	9	46.7	21.4	6	NS
F	Stress	630.9	602.4	8	558.6	346.9	4	NS	0.7	0.4	8	0.6	0.3	4	NS	12.2	7.6	8	9.2	6.1	4	NS	38.2	18.7	8	44.4	35.2	4	NS

Within sex protein analysis (2-way ANOVAs) from pre-processed samples remaining from mRNA extraction from the amygdala. Select targets were analyzed: corticotropin releasing hormone (CRH); interleukin-1 beta (IL-1β), FK506-binding protein 51 (FKBP5), and NOD-like receptor family pyrin domain-containing protein 3 (NLRP3). Prenatal alcohol exposure (PAE) resulted in elevated CRH protein in male offspring independent of stress exposure (*F*_(1,18)_ = 4.617, *p* < 0.05), with a strong trend toward a significant elevation of IL-1β protein in male offspring with PAE independent of stress exposure (*F*_(1,19)_ = 4.132, *p* = 0.05) was revealed. NS, not significant; ^*^p < 0.05, ^#^*p* = 0.05. ^*^ Main effect of PAE: *F* (1,18) = 4.617, *p* = 0.045. ^#^ Main effect of PAE: *F* (1, 19) = 4.132, *p* = 0.056.

AVP, arginine vasopressin; GAS5, growth arrest specific 5; FKBP4, FK506-binding protein 52; GFAP, glial fibrillary acid protein; TMEM119, transmembrane protein 119; HMGB1, high mobility group box 1; TLR4, toll-like receptor 4; NFKBIA, NF-κB inhibitor alpha; NLRP3; IL1B; TNF, tumor necrosis factor alpha; CCL2, C–C motif chemokine ligand 2; IL1R1, interleukin-1 receptor 1; IL10, interleukin-10.

## Discussion

Accumulating evidence supports the adverse impact of PAE on CNS function that often goes unnoticed until a secondary challenge occurs after birth and well into adulthood, typically from direct immune activation or from direct peripheral tissue damage. Significant PAE-induced susceptibility of the CNS to dysregulation may involve sensitized inflammatory cytokines/chemokines and transcriptional factors (Drew et al., 2015; Bodnar et al., 2016, 2020; Terasaki and Schwarz, 2016; Noor et al., 2017, 2020; Sanchez et al., 2017, 2019). The current report extends these observations by demonstrating that a secondary challenge can include non-tissue damaging stressors occurring in early life, and supports that both neuroimmune and neuroendocrine dysregulation may underlie early programing that leads to adult CNS vulnerabilities. In support of prior studies, a striking demonstration is made in the current report that the amygdala reveals significant mRNA upregulation of the stress-associated factors CRH and AVP (Figure 1A; Table 2), disruptors of glucocorticoid receptor signaling GAS5 and FKBP5 (Figure 2B; Table 2), the microglial activation marker transmembrane protein 119 (TMEM119; Figure 3B; Table 2), and factors that initiate or are induced downstream of TLR4 activation (Figure 4B; Table 2) by PAE in offspring treated with a single session of maternal separation stress at PND10.

Other proinflammatory factors measured in the amygdala that are not necessarily dependent on TLR4 activation, but can impact TLR4 downstream effects and are critical for mediating aspects of innate immune proinflammatory progression are altered in females as a consequence of stress (TNF), or with disparate IL1R1 stress-response changes between SAC and PAE (Figure 5B and Table 3). IL-10, as an endogenous anti-inflammatory cytokine showing compensatory upregulation upon inflammatory cytokine signaling, is robustly increased in females with PAE that are also exposed to maternal separation stress (Figure 5B; Table 3).

In the hypothalamus, blunted CCL2 (in males), IL1R1 and IL-10 (in females) with strong trends toward blunted TNF levels as a consequence of PAE are observed (Figure 5C; Table 3). The extent of low hypothalamic mRNA neuroimmune factors under basal conditions and blunted in response to stress in offspring with PAE consistently extends to a large number of proinflammatory indicators, as shown by blunted glial activation (astrocytes and microglia; Figure 3C; Table 3), factors associated with the TLR4 signaling cascade (HMGB1, TLR4, NFKBIA, and NLRP3; Figure 4C; Table 3), that were revealed predominantly in female offspring. Even strong trends in reduced CCL2 expression are shown in females (Figure 5C). The observed data from hypothalamic tissue are remarkably consistent, and while speculative, suggest that the underlying hypothalamic process may involve an exaggerated stress *hypo*responsive period. That is, during later life stressors, the hypothalamus may be reprogramed as less responsive to elevated circulating glucocorticoids, with diminished glucocorticoid receptor function until elevated glucocorticoid levels at longer-durations are achieved before the activation and downstream impact by glucocorticoids occur.

While an earnest attempt was made to measure protein levels of CRH, FKBP5, NLRP3 and IL-1β from the amygdala, only CRH protein from males revealed significant increases relative to controls without PAE (Table 4). Considering the pattern of CRH mRNA together with protein CRH in the male amygdala, the data suggest that protein levels retrieved from these samples are too low to achieve interpretable results. Moreover, due to loss of material, large discrepancies in samples sizes for various targets resulted, and the resultant data analyses are limited in this respect. No additional changes in protein levels were observed.

The amygdala is uniquely positioned to regulate responses to psychogenic stressors, as cortical association areas project to the amygdala, and in turn, the amygdala influences the function of critical stress-integrative brain areas such as the hippocampus and hypothalamus through direct amygdala-hippocampal (Stefanacci et al., 1996; Leal et al., 2017; Zheng et al., 2019) and amygdala-hypothalamic circuitry (Alexander et al., 2012). In addition, the amygdala is characterized as the fundamental brain area supporting fear-related behaviors such that a perceived threat is immediately processed as dangerous (Murison, 2016; Godoy et al., 2018) with amygdaloid activation being important for emotional retrieval and analysis of any given stressor. The amygdala can directly stimulate components of brain stress-response circuits via CRH neuropeptide release in (a) an autocrine manner within the amygdala and also (b) at more distant sites such as in the hippocampus (Tsigos et al., 2000). Notably, CRH peptidergic neurons in the amygdala are activated by glucocorticoids and upon activation, lead to the stimulation of the stress circuitry and fear/anxiety (Kolber et al., 2008). The amygdala-hippocampus circuit is bidirectional, and under normal conditions, the hippocampus can exert a tonic and evoked inhibitory effect on CRH neurons of the amygdala. Indeed, the hippocampus plays an important role in shutting off the HPA stress response [(Tsigos et al., 2000) for Review].

In light of this background concerning hippocampal influences on amygdala function, one aspect of the CNS stress-response system examined by the current report is the potential impact that PAE exerts on general amygdaloid responses to a single, acute stress (maternal separation). The current data demonstrate pronounced changes in mRNA expression of CRH and AVP, both of which are significantly elevated in the amygdala (Figure 1A). Unexpectedly, these changes are observed from tissue derived from females with PAE and not from males.

Under non-pathological conditions, the ventral hippocampus has been characterized to influence hypothalamic responses resulting in activation of hypothalamic subnuclei. The present data show that blunted AVP mRNA expression is observed in males (Figure 1B), which may be a consequence of dysregulated ventral hippocampal influences on hypothalamic subnuclei (e.g., lateral hypothalamic area) by PAE. However, meaningful mRNA changes of CRH in the hypothalamus were not observed in neither males nor females.

The current data also show that not only are stress-associated factors elevated in the amygdala following PND10 maternal separation, but also factors that disrupt glucocorticoid receptor signaling. Glucocorticoid receptors are a key effector element of the HPA stress response (De Kloet et al., 1988; Oakley and Cidlowski, 2013). Glucocorticoid receptors interact with factors that function to fine tune the HPA stress response, thereby regulating glucocorticoid receptor signaling or its sensitivity. GAS5 is a long non-coding RNA expressed throughout the body originally found to have tumor suppressor activity by arresting the growth cycle of stressed cells, but also promotes apoptosis and acts to remove micro-RNA, ultimately decreasing glucocorticoid-dependent transcription of genes; it essentially acts as a decoy glucocorticoid response element (GRE) within the cell nucleus (Petta et al., 2016; Yu and Hann, 2019). The current report reveals that transcriptional expression of GAS5 is elevated in the male hippocampus, and to a much greater degree, in the female amygdala of PAE offspring following maternal separation stress (Figures 2A,B). The data support the possibility that glucocorticoid receptor signaling is reduced only in PAE offspring, which is unmasked by the secondary challenge of an acute maternal separation stress in females.

Interestingly, prenatal arsenic exposure induced sex-specific differences in the expression of GAS5 mRNA bound to the glucocorticoid receptor in the developing telencephalon (gestational days 14–18) (Caldwell et al., 2018), which includes the neocortex and the hippocampus (Hebert and Fishell, 2008). Arsenic has been characterized to impact the glucocorticoid and HPA axis system (Tyler and Allan, 2014). The data by Caldwell and colleagues (Caldwell et al., 2018) demonstrate that arsenic-exposed female telencephalon revealed relatively lower bound GAS5-to-glucocorticoid receptor compared to males, suggesting that males undergo reduced control over telencephalic glucocorticoid signaling and/or regulation during this critical gestational period. That is, during late gestational periods, glucocorticoids facilitate fetal neuronal maturation, axon remodeling and cell survival (De Kloet et al., 1988). Note that in the current report, GAS5 in female hippocampus with PAE is significantly lower (Figure 2A; Table 3) compared to the opposite trend observed in male hippocampus. While speculative, reduced glucocorticoid receptor signaling during this late gestational period may impact postnatal corticolimbic development resulting altered responses to stress during later life, particularly in males.

However, while lower, presumably protective GAS5 mRNA levels are demonstrated in the female hippocampus from PAE, a striking elevation of GAS5 in the female amygdala with PAE is revealed upon stress exposure (Figure 2B). The data are additionally striking in that no significant alterations are observed in the male amygdala of any glucocorticoid receptor signaling regulator examined in the current report (GAS5, FKBP5, and FKBP4), suggesting that adverse stress responses between males and females are regulated by different corticolimbic areas. Prior reports have demonstrated sex differences in glucocorticoid dysregulation involving hippocampal pathways, and a discussion of the data considers the possibility that PAE impacts glucocorticoid signaling in different pathways of the female brain (Caldwell et al., 2018). Our current data support and extend the differential impact of PAE between male and female limbic pathways, and especially following stress.

Glucocorticoid receptors are present in the cytoplasm in a complex with other proteins including chaperone proteins and FKBP51 (encoded by FKBP5) (Schiene-Fischer and Yu, 2001; Oakley and Cidlowski, 2013) that ultimately reduces glucocorticoid receptor signaling (Zannas et al., 2016). In further support that PAE dysregulates glucocorticoid receptor signaling, elevated levels of FKBP5 are observed in the hippocampus and, again to a greater magnitude, in the amygdala following stress (Figures 2A,B; Table 3). Significantly increased FKBP5 may act to further reduce the affinity of glucocorticoid receptor-glucocorticoid ligand binding, as has been previously considered (Schiene-Fischer and Yu, 2001). An additional mechanism by which FKBP51 may alter glucocorticoid receptor signaling is via FKBP51’s direct interaction with the IKK complex (scaffold and isomerase activity) that ultimately increases NF-kB activation (Romano et al., 2015). NF-kB is a pivotal transcription factor that regulates the expression of several potent proinflammatory cytokines such as IL-1β, which is increased in the hippocampus and amygdala following stress and reduces glucocorticoid receptor signaling (Sugama et al., 2011; Wohleb et al., 2011).

The current study additionally examined the transcriptional expression of FKBP52, encoded by the FKBP4 gene, as it promotes high affinity binding of glucocorticoid receptors with glucocorticoids through interactions with microtubule proteins and thus facilitates the nuclear translocation of this complex (Schiene-Fischer and Yu, 2001; Davies et al., 2002). While FKBP51 and FKBP52 compete for binding to the glucocorticoid receptor in the cytoplasm, evidence demonstrates that in the presence of both FKBP52 and FKBP51, the glucocorticoid receptor preferentially associates with FKBP51 (Barent et al., 1998). The data from the current report demonstrate that elevated transcriptional expression of FKBP4 is present in male hippocampus from offspring with PAE and maternal separation stress (Figure 2A; Table 3), and to a large degree in female amygdala from offspring with PAE and stress exposure (Figure 2B; Table 3). Given that elevated FKBP51 binds glucocorticoid receptors reducing glucocorticoid ligand binding and preferential glucocorticoid receptor-FKBP51 occurs in the presence of FKBP52, an examination of the FKBP5:FKBP4 ratio was additionally performed. The data show that significantly elevated FKBP5:FKBP4 is present only in the hypothalamus of PAE female offspring, further supporting the possibility that PAE reduces glucocorticoid receptor signaling. Together, the observation that increased GAS5 with concurrent elevations of FKBP5 in the hippocampus, and to a much greater degree, in the amygdala of PAE offspring following maternal separation stress support the possibility that PAE dysregulates CRH/AVP actions and glucocorticoid receptor signaling in the amygdaloid-hippocampal circuit response to early life stress.

Evidence is mounting that adverse CNS function from PAE offspring is due to elevated expression of brain proinflammatory cytokines, chemokines, and increased activation of related immune receptors and glia (Drew and Kane, 2014; Drew et al., 2015; Noor and Milligan, 2018). Further, the possibility that dysregulated neuroimmune function may adversely impact the brain stress-response and neuroendocrine system has been emerging for a number of years (Weinberg, 1989; Zhang et al., 2005; Hellemans et al., 2008, 2010; Bodnar et al., 2016; Raineki et al., 2017; Lam et al., 2018). Given cytokines are now characterized to act as important contributors to healthy brain development (Smith et al., 2007; Jakovcevski et al., 2009; Stephan et al., 2012), alterations in the levels and regulation of a number of immune-related factors can wreak havoc on brain function, including the brain stress-response system. The current report extends this prior work demonstrating that significantly increased amygdaloid microglial activation is present in female offspring with PAE following maternal separation stress (Figure 3B; Table 3), suggesting that the consistent interaction of PAE and early life stress on glucocorticoid signaling blockers, and proinflammatory signaling factors may be mediated by microglial activation. However, not all brain areas examined demonstrated consistent microglial activation, as blunted hypothalamic microglial activation is observed in female PAE offspring (Figure 3C; Tables 2, 3), with no such changes occurring in male PAE offspring.

Activated TLR4 results in the synthesis of a number of proinflammatory factors even following non-tissue damaging stressors. For example, non-physiological stress (i.e., maternal separation) can induce the release of classic immune danger-associated molecular patterns (DAMPs) such as the high-mobility group box 1 (HMGB1) independently of cell necrosis (Fleshner et al., 2017; Johnson et al., 2019). HMGB1 released from neurons, glia and immune cells activates TLR4. Following TLR4 activation by DAMPs, intracellular signaling mediated by myeloid differentiation factor 88 (MyD88) that ultimately induces activation of the NFkB transcription factor that initiates the synthesis of a number of proinflammatory cytokines including tumor necrosis factor-α (TNF-α, encoded by TNF), and precursor proteins pro-IL-1β (encoded by IL1B), pro-caspase-1 and the inflammasome, Nod-like receptor family pyrin domain containing 3 (NLRP3). Activated NLRP3 interacts with pro-caspase-1 to form the NLRP3 inflammasome protein complex, allowing for caspase-1 activation and consequent conversion of pro-IL-1β to mature, active IL-1β (Liu et al., 2017). Termination of active NFkB transcription is based on NFkB-dependent gene transcription of I kappa B-alpha (IkBa), an inhibitory subunit encoded by the NFKBIA gene (Bottero et al., 2003). Thus, quantification of NFkB activity is best captured by assessing gene synthesis encoding the protein IκB-α because induction of NFkB is reliably followed by NFKBIA mRNA synthesis, and a reduction in NFkB transcriptional activity is reflected in a reduction in NFKBIA mRNA. Notably, stabilization and accumulation of NFKBIA does not occur, which allows for the real time examination of NFKBIA mRNA levels to indirectly assess NFkB activity (Bottero et al., 2003).

In support of this background, the data from the current report reveals a striking increase in factors characterized to be induced following TLR4 activation predominantly in amygdala of female PAE offspring in response to maternal separation stress (Figure 4B; Table 3). Compared to prenatal control exposed offspring, reliable increases in mRNA for HMGB1, TLR4, NFKBIA, NLRP3 as well as IL1B are observed in the amygdala of offspring with PAE in response to stress, suggesting a primed proinflammatory state is created by PAE that is triggered by the acute stressor. It is notable that the significant alterations in these factors are observed predominantly in females rather than males. However, significant TLR4 and NLRP3 mRNA increases are observed in hippocampus of male PAE offspring regardless of stress exposure (Figure 4A; Table 3), while no changes in TLR4-associated factors were present in the male amygdala, consistent with a lack of astrocyte and microglial activation. Further, the hypothalamus from male offspring with PAE reveals blunted astrocyte activation as well as a non-significant trend toward blunted microglial activation. Combined, the data suggest that during early postnatal life, stress-induced increases in TLR4-related factors are observed different limbic structures between males and females, with some factors altered in the male hippocampus, and a large majority of these factors altered in female hippocampus and amygdala. One possibility may be that with regard to neuroimmune dysregulation, males may have greater protection against stress-induced neuroimmune dysregulation, but may be more vulnerable to stress-induced glucocorticoid receptor insensitivity.

The pathophysiology of PAE that is mediated by proinflammatory factors that can influence TLR4 activation and downstream cellular signaling. These factors include TNFα, CCL2, IL-1R1 alterations, and compensatory production of the and the anti-inflammatory cytokine, IL-10 (Noor and Milligan, 2018). Brain TNFα and CCL2 are upregulated as a consequence of TLR4 activation following alcohol exposure (Alfonso-Loeches et al., 2010; Montesinos et al., 2016; Pascual et al., 2017), and other recent reports suggest that PAE leads to suppressed anti-inflammatory IL-10 expression in peripheral and central nervous tissue (Noor et al., 2017; Sanchez et al., 2019; Noor et al., 2020). Notably, IL-10 is well-characterized to limit the actions of IL-1β, TNFα and CCL2, in addition to a number of other proinflammatory factors (Foey et al., 1998). The data from the current report demonstrate elevated IL1R1 mRNA is unmasked in the hippocampus of female offspring with PAE due to a stressor, in support of IL-1R1 brain elevations from inflammatory insults (Andre et al., 2005). Hippocampal IL10 mRNA elevations are observed only in stressed PAE male offspring (Figure 5A; Tables 2, 3), suggesting that IL-10 in males may be one mechanism conferring protection from stress-induced neuroimmune alterations.

Sex-specific outcomes as a consequence of PAE have been documented demonstrating sexually dimorphic effects of activity-dependent hippocampal glucocorticoid receptor protein levels (Caldwell et al., 2018), as well as HPA activity in PAE offspring (Weinberg et al., 2008; Lam et al., 2018; Lussier et al., 2021). While the inflammatory effects between PAE and/or stress on placental cytokines are similar (Ruffaner-Hanson et al., 2022), the levels of these inflammatory factors are distinctly different in male versus female fetuses (Terasaki and Schwarz, 2016). While speculative, the current data demonstrating consistent elevations in stress-associated and immune-related factors in the amygdala of PAE females, and blunted hypothalamic expression of many of these same markers suggests that PAE predisposes females to dysregulated limbic stress responses (e.g., CRH, AVP, GAS5, FKBP5 and FKBP4) as a consequence of altered glial and TLR4-assoicated factors. Interestingly, females often demonstrate an increased incidence of immune disorders and increased brain proinflammatory cytokine gene activation in animal models also expressing depressive-like behaviors (Whitacre, 2001; Tonelli et al., 2008). Thus, the sensitized female limbic pathways from PAE due to underlying alterations in the stress and neuroimmune axis may be of particular relevance to understanding the development of mood disorders later in life in individuals with an FASD (Hellemans et al., 2008; Hagan et al., 2016; van Bodegom et al., 2017; Wang et al., 2019). If dysregulated limbic cytokine expression is ultimately found to be critical in the neuropathology of mood disorders, the intriguing possibility emerges that treatment strategies can be identified that target re-balancing the brain cytokine milieu in individuals living with FASD.

In conclusion, our sex-specific findings of robustly elevated transcriptional expression of key stress-related neuropeptides as well as TLR4 signaling pathway factors in the female amygdala that is unmasked by early postnatal maternal separation stress provides new insight into how PAE may remodel developing brain areas responsible for integrating the stress response during early and adult life. The data further reveal that PAE *per se*, is insufficient to alter basal transcription of stress-related neuropeptides and TLR4 neuroimmune factors, but instead, changes in gene expression are unmasked only following stressor exposure. Thus, together the results support our hypothesis that PAE sensitizes key stress-integrative limbic circuits such that significant exaggerated responses in the amygdala and blunted responses in the hypothalamus are unmasked due to early life stress. Prior work demonstrates a unique cytokine profile such as IL-1β and TNFα, from PAE occurs in the hippocampus, prefrontal cortex and the hypothalamus depending on early postnatal age, with blunted patterns of expression observed in the hypothalamus (Bodnar et al., 2016). This work suggests that even subtle changes in brain cytokine expression levels may act to alter the course of normal brain development. It is possible that in the PAE brain, even minor stressors during early postnatal life may be sufficient to exert enduring developmental changes.

## Data availability statement

The original contributions presented in the study are included in the article/supplementary material, further inquiries can be directed to the corresponding author.

## Ethics statement

The animal study was reviewed and approved by University of New Mexico Animal Care and Use Committee.

## Author contributions

CR–H performed all the real-time PCRs and statistical analysis, and prepared the first draft of the manuscript. AF-O performed the protein ELISAs and statistical analyses. MS participated in tissue collection. KC and AA contributed to conception and design of the study, generated the mouse model of prenatal alcohol exposure, and collected tissue. EM contributed to conception and design of the study, participated in collection of tissues, assisted in statistical analyses, and prepared the manuscript. DS and CV supported the generation of the mouse model of prenatal alcohol exposure. SN contributed to tissue collection, real-time PCR data collection, statistical analysis and edited the manuscript. All authors contributed to the article and approved the submitted version.

## Funding

The experiments conducted to generate this manuscript were funded by the NIH NIAAA 2P50 AA022534, and R01-AA025967.

## Conflict of interest

The authors declare that the research was conducted in the absence of any commercial or financial relationships that could be construed as a potential conflict of interest.

## Publisher’s note

All claims expressed in this article are solely those of the authors and do not necessarily represent those of their affiliated organizations, or those of the publisher, the editors and the reviewers. Any product that may be evaluated in this article, or claim that may be made by its manufacturer, is not guaranteed or endorsed by the publisher.

## Footnotes

^1^https://www.graphpad.com/quickcalcs/grubbs1/
